# Promoting Employee Health Through an American Cancer Society Program, The CEOs Challenge, Washington State, 2013–2015

**DOI:** 10.5888/pcd12.150381

**Published:** 2015-12-17

**Authors:** Jeffrey R. Harris, Amanda T. Parrish, Marlana Kohn, Kristen Hammerback, Becca McMillan, Peggy A. Hannon

**Affiliations:** Author Affiliations: Amanda T. Parrish, Marlana Kohn, Kristen Hammerback, Peggy A. Hannon, Health Promotion Research Center, School of Public Health, University of Washington, Seattle, Washington; Becca McMillan, American Cancer Society Great West Division, Seattle, Washington.

## Abstract

**Introduction:**

Evidence-based practices in the workplace can increase levels of healthy eating, cancer screening, physical activity, and tobacco cessation but are underused, even in large workplaces. This report summarizes an evaluation of the first year of The CEOs Challenge, a program developed by the American Cancer Society to promote implementation and maintenance of health-promoting, evidence-based workplace practices by large companies.

**Methods:**

Use of 17 evidence-based practices by 17 companies in the Washington State Chapter of the American Cancer Society’s CEOs Against Cancer network was assessed via survey and scored from 0 to 100. Companies received a written report of their baseline performance, followed by at least quarterly consultations with American Cancer Society staff members trained to assist in implementation of these practices. Follow-up performance was measured at 1 year.

**Results:**

At baseline, implementation scores were 54.8 for cancer screening, 46.5 for healthy eating, 59.8 for physical activity, and 68.2 for tobacco cessation. At follow-up, scores increased by 19.6 for cancer screening, 19.4 for healthy eating, 16.0 for physical activity, and 9.4 points for tobacco cessation.

**Conclusion:**

The CEOs Challenge is a promising approach to chronic disease prevention via the workplace. It brings together one of the nation’s largest health-promoting voluntary agencies with the nation’s largest employers to promote evidence-based practices targeted at the most common causes of disease and death. The program increased the adoption of these practices and was well-accepted.

## Introduction

Chronic diseases are rampant in the United States (1), and workplace health promotion programs can play an important role in their prevention and control (2). Most American adults work, and their workplaces provide an organizational, physical, and social setting for implementing evidence-based practices (EBPs) that promote health (3). Large workplaces are particularly well-suited to workplace health promotion because of their financial and staff resources (3), yet large workplaces underuse EBPs (4,5).

To disseminate health-promoting EBPs to large workplaces, the American Cancer Society (ACS) and the University of Washington’s Health Promotion Research Center (HPRC) developed the Workplace Solutions program (5). The EBPs measured and supported in Workplace Solutions are largely derived from the Guide to Community Preventive Services (6) and promote healthy eating, physical activity, tobacco cessation, and screening for breast, cervical, and colorectal cancers. In a pre–post evaluation, Workplace Solutions significantly increased implementation of these EBPs by large workplaces in the Pacific Northwest. Soon after, ACS began a national program in which its staff (7,8) delivered Workplace Solutions to more than 1,700 workplaces employing 6.9 million people in 42 states (9). Although widespread delivery of this program was encouraging, interviews with ACS staff (K.H., unpublished data, 2012) indicated that ongoing contact after initial delivery to participating companies was minimal, little follow-up assessment of EBP implementation took place, and the degree of EBP maintenance was unknown.

Meanwhile, ACS developed its Chief Executive Officers (CEOs) Against Cancer network, which organizes executives of large companies (companies that have ≥5,000 employees) into regional chapters to take action in cancer prevention and control (10). The network is active in 17 US cities and has 570 member companies. In 2013, members of the Washington State Chapter (hereinafter referred to as the Chapter) of CEOs Against Cancer decided to focus their collective effort on health promotion among employees in their own companies, and Chapter leadership collaborated with ACS staff and HPRC researchers to adapt and update Workplace Solutions for this purpose.

The updated program was called The CEOs Challenge. CEOs provide leadership that is crucial to the success of workplace health programs (3) and often compete with their peers at other companies. The CEOs Challenge was designed to promote implementation and maintenance of workplace health promotion EBPs through annual assessments of EBP implementation and support from ACS staff members via quarterly check-ins. A scoring system that allows CEOs to review their implementation progress annually and compare their progress with that of their peer organizations was developed for the assessments. This report summarizes an evaluation of the first year of The CEOs Challenge.

## Methods

The Chapter comprises 26 active member companies, employing a total of 348,527 people. Executives (CEOs or, when companies are headquartered elsewhere, regional chief executives) gather at Chapter meetings 3 times per year. Although Workplace Solutions was designed for companies with 5,000 employees or more, the companies in the Chapter vary widely in size, and all were welcome to participate in The CEOs Challenge. The October 2013 meeting served as the kickoff. At this meeting, executives learned about the program and, if they chose, made an official commitment to participate. The CEOs Challenge was presented as a continuous program, with an annual cycle, to support companies in their efforts to improve employee health.

Because company representatives reported on company-level activities, the University of Washington’s institutional review board did not consider these activities subject to review for human subjects research.

### Assessment of EBP implementation

The CEOs Challenge is a unique intervention in that assessment measures are a key part of the intervention and are reported to participants. 

**Baseline survey.** Participating executives delegated a company representative, almost always from Human Resources, to serve as manager of The CEOs Challenge program. At baseline, this manager completed the 41-item Workplace Solutions survey (5) assessing company demographics and implementation of best EBPs aimed at reducing cancer and chronic disease among employees. We encouraged managers to collect information from the person most knowledgeable about a particular best practice (for example, the lead person on health insurance benefits or the lead person on wellness programs). For local or regional offices of companies headquartered outside of Washington State, we instructed managers to report on demographics and best-practice implementation in their region. Of the 26 companies in the Chapter at baseline data collection, 21 completed baseline assessments between November 2013 and June 2014. Four companies disengaged from the program after baseline data collection. Of the 17 remaining companies, 15 were headquartered in Washington State. Thirteen companies designated managers from their Human Resources departments.

**Score reports.** We measured implementation of 17 EBPs in 4 categories: cancer screening, healthy eating, physical activity, and tobacco cessation. The ACS also added 4 additional best practices under a fifth category, corporate philanthropy. On the basis of data provided via the survey, we used explicit criteria to score each of the 21 best practices as not implemented, partially implemented, or fully implemented. For example, a company with no physical activity program would receive “not implemented” as a score for this best practice, a company with an existing program that did not meet all best-practice criteria (for example, the program was not group based or did not include individual goal setting) would be scored as “partially implemented,” and a company with a program meeting all best-practice criteria would be scored as “fully implemented.” Individual best-practice scores in each of the 4 health-related categories were totaled and weighted with a multiplier to provide a numerical score (0–100). Category scores were averaged to provide an overall company score (also 0–100). Category scores were presented to executives and managers in an intuitive bar-chart format (Figure), whereas tables were provided to show the implementation status of best practices in a given category.

**Figure:**
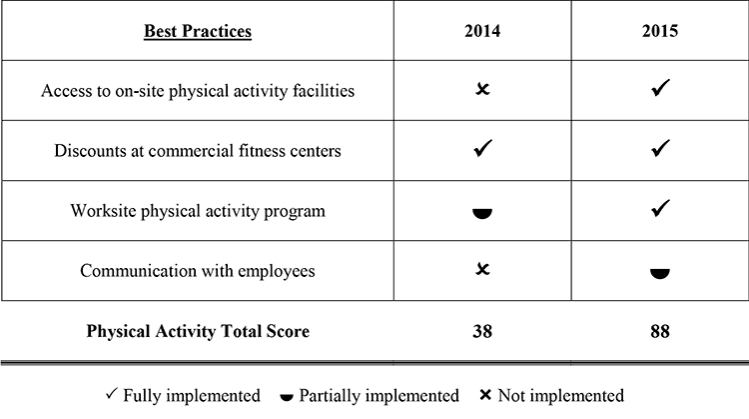
Example of a score report, showing the 4 best practices for promoting physical activity, sent to a company participating in the American Cancer Society’s CEOs Challenge, Washington State, 2013–2015. **Table:** 

Best practice	2014	2015
Access to on-site physical activity facilities	Not implemented	Fully implemented
Discounts at commercial fitness centers	Fully implemented	Fully implemented
Worksite physical activity program	Partially implemented	Fully implemented
Communication with employees	Not implemented	Partially implemented
Physical activity total score	38	88

**Company engagement.** ACS staff members held an in-person meeting with the manager of each participating company to present their CEOs Challenge score report and select health promotion focus areas for the coming year. At a second in-person meeting, ACS staff members made recommendations for making progress in each category. Further in-person meetings were held at least quarterly, but no more than monthly, and were augmented by support through emails and telephone calls to aid implementation of best practices. In addition, ACS staff members met 1 to 3 times per year with the company executive to ensure leadership commitment. We provided CEOs Challenge updates and reminders to executives at the thrice-yearly Chapter meetings.

**Annual follow-up.** Company managers in 17 companies participated in a follow-up survey approximately 12 months after baseline; all surveys were completed by May 2015. The survey items and score calculation were identical to those at baseline, supplemented by 4 qualitative program-satisfaction items (“How would you describe your experience with this program, over the past year?”; “Which aspects of The CEOs Challenge did you find most useful or helpful?”; “What would make The CEOs Challenge work better for your organization?”; “Do you have any final thoughts, or is there anything else we should know?”). After survey completion, new score reports were presented to companies, new or ongoing health promotion focus areas were identified for the coming year, and company engagement continued in the same way as in the first year. Follow-up score reports highlighted changes in best practices during the first year. Moving forward, the intent is to continue conducting annual follow-up surveys and delivering reports to participating companies.

**Recognition.** Various forms of public recognition were used. At baseline, we aggregated company scores and presented them to executives at a Chapter meeting, allowing each company to compare its status against other participating companies. At each Chapter meeting during the year, executives were asked to share success stories. At follow-up, we again aggregated company data and presented Chapter-wide scores. Each company was thus able to see its own progress during the year and to compare its progress with that of the Chapter as a whole.

Company managers also received recognition. All were invited to participate in a half-day CEOs Challenge Wellness Workshop organized by ACS. Managers of particularly successful programs were invited to make a presentation, and awards of recognition were given to all participants.

### Data analysis

We analyzed data only for the 17 companies that had complete baseline and follow-up data. Descriptive statistics were computed for the characteristics of employees in participating companies. A 2-sided, paired *t* test was run to assess whether average changes in aggregate overall company best-practice scores from baseline to 12-month follow-up were different from zero. A signed rank test was also performed as confirmatory analysis in response to the skewness of change-score data. The aggregate scores for best-practice categories were presented descriptively without *P* values, because Bonferroni adjustments for multiple measurements were underpowered and statistically unreliable in this sample. Analysis was completed using Stata version 11.2 (StataCorp LP).

Data from qualitative program-satisfaction items were analyzed by 3 team members: one team member (M.K.) systematically reviewed the data to derive and classify themes and subthemes. Two other team members (A.T.P. and J.R.H.) reviewed the data and confirmed themes and subthemes.

## Results

Median company size was 3,744 employees, and mean employee salary was $56,200 (Table 1). Most employees were aged 25 to 54 (72.2%), white (70.3%), and non-Hispanic (94.0%). More than half (58.5%) were women. Not all companies were able or willing to provide detailed demographic data about their workforce.

**Table 1:** Characteristics of Employees in Companies (n = 17) Participating in the American Cancer Society’s CEOs Challenge, Washington State, 2013–2015^a^

Characteristic	No. of Employers That Provided Data	Value
**Employees, median (range), n**	17	3,744 (32–251,196)
**Annual salary, mean (SD), $**	12	56,200 (19,822)
**Sex, mean (SD), %**		
Male	15	41.5 (16.3)
Female		58.5 (16.3)
**Race, mean (SD), %**		
African American	11	6.1 (5.3)
White		70.3 (18.6)
Asian/Pacific Islander		10.9 (7.1)
Other		12.8 (17.7)
**Hispanic/Latino, mean (SD), %**	11	6.0 (5.0)
**Age, mean (SD), %**		
18–24 y	13	7.2 (7.6)
25–54 y		72.2 (7.2)
55–64 y		17.7 (5.1)
≥65 y		2.8 (1.1)

Abbreviations: CEO, chief executive officer; SD, standard deviation.

a Data are from baseline surveys of companies participating in The CEOs Challenge that also completed follow-up surveys. Some employers were not willing or able to provide detailed demographic information about their employees.

At baseline, companies performed best in tobacco cessation best practices; this performance was driven by high levels of coverage for nicotine-replacement therapy and tobacco-cessation counseling (Table 2). Companies struggled most with healthy eating best practices, scoring least well in competitive pricing of healthy foods and policies for providing healthy options at meetings or employee events. Scores varied by company across categories. Scores for healthy eating and physical activity ranged from 0 to 100; for tobacco cessation, 20 to 100; and for cancer screening, 17 to 100. Sixteen companies participated in some form of ACS-affiliated corporate philanthropy.

**Table 2:** Mean Baseline, Follow-Up, and Change Scores for Workplace Health Promotion Best Practices Recommended to Companies (n = 17) Participating in the American Cancer Society’s CEOs Challenge, Washington State, 2013–2015

Category and Best Practice	Mean Baseline Score	Mean Follow-Up Score	Change in Score^a^
**Cancer screening**			
Company uses systems approaches that increase screening	41	71	29
Company has policies that support screening during work hours	88	100	12
Company communicates with employees about cancer screenings	35	53	18
Overall for category, mean (range)	54.8 (17 to 100)	74.4 (33 to 100)	19.6 (–17 to 67)
**Healthy eating**			
Healthy foods are available	75	93	18
Company subsidizes/competitively prices healthy food	29	43	13
Nutritional content of foods is posted, or healthy food choices are labeled	35	79	43
Company worksites adhere to catering guidelines	18	47	29
Company communicates with employees about the importance of healthy eating and nutrition	68	74	6
Overall for category, mean (range)	46.5 (0 to 100)	65.9 (0 to 100)	19.4 (0 to 80)
**Physical activity**			
Company provides access to on-site physical activity facilities	53	71	18
Company provides negotiated discounts/incentives for employee membership at commercial fitness centers	76	76	0
Company provides worksite-based physical activity program	57	79	23
Company communicates with employees about the importance of physical activity	47	76	29
Overall for category, mean (range)	59.8 (0 to 100)	75.7 (0 to 100)	16.0 (–12 to 83)
**Tobacco cessation**			
Tobacco use is banned at worksites, on company property, and in company vehicles	65	74	9
Company fully covers non-nicotine tobacco cessation medications with no out-of-pocket expense	66	79	14
Company fully covers nicotine-replacement therapy medications with no out-of-pocket expense	81	85	4
Company fully covers tobacco cessation counseling with no out-of-pocket expense	82	85	3
Company communicates with employees about tobacco cessation, cessation medication, and cessation counseling	53	65	12
Overall for category, mean (range)	68.2 (20 to 100)	77.6 (30 to 100)	9.4 (–10 to 50)
**Corporate philanthropy^b^**			
Company takes a leadership role or sponsors employee participation in ACS cause-related events	76	76	0
Company allows employees to take advantage of ACS volunteer opportunities on company time	65	59	−6
Company offers employees donation opportunities	47	53	6
CEO leads a unique fundraising project such as a capital campaign, fundraising to support research, or a distinguished event	50	65	15
Overall for category, mean (range)	60.3 (0 to 100)	63.2 (0 to 100)	2.9 (–50 to 50)
**Overall best practice score (excluding corporate philanthropy)**	**57.3 (23 to –87)**	**73.4 (26 to 91)**	**16.1 (–2 to 56)**

Abbreviations: ACS, American Cancer Society; CEO, chief executive officer.

a All values are mean unless otherwise indicated. Mean follow-up score minus mean baseline score may not equal value in column for mean change in score (a difference of 1) because of rounding.

b Company implementation of corporate philanthropy best practices was included in the scores presented to participants in The CEOs Challenge, but was not included in our calculation of change in implementation of health-related best practices between baseline and follow-up.

Scores improved between baseline and 12-month follow-up (Table 2), with aggregate Chapter improvements in all 4 categories. The mean absolute change in aggregate overall company best-practice score from baseline to follow-up was 16.1 points (*P* < .001). Cancer screening practices improved 19.6 points with improvements in screening reminders in health insurance contracts. Healthy eating scores increased an average of 19.4 points, with companies increasingly posting nutritional content, labeling healthy choices, and adhering to healthy catering guidelines for meetings and events. Improvements in physical activity and tobacco cessation best practices were driven by improved communications with employees. Corporate philanthropy efforts were strong at baseline and improved slightly at 12-month follow-up.

At 12-month follow-up, company experience with the program was positive, according to the qualitative portion of the survey. Managers noted that the health promotion focus of the program built on existing company initiatives and offered insight into areas where companies could expand and improve wellness efforts. “This has been a great eye-opening program to our company. It has opened our eyes to things that we really need to be working on, as well as what we do well” (Company C). “The program helped us identify where we could expand our wellness offerings and provided us the tools/support to do so” (Company F).

Managers found most helpful the direct support from the ACS team and the ability to see how their own company compared with peer companies: “The ACS team was the best part of [The CEOs Challenge]. They helped suggest ways to improve [our] employee health and provided resources to help make it happen” (Company H). “We tend to be a little competitive, so establishing a challenge like this with other large local companies was fun for us” (Company L). “We liked seeing where other companies are excelling and even struggling. It helps to give us a . . . direction of where we need to be focusing” (Company C).

Managers wanted additional online resources for employees, regular updates to leadership to aid best-practice improvements, and help in focusing on a particular aspect of cancer prevention, such as cancer screening. One manager asked to have more interaction between the executives in the Chapter and the human resources staff members who run their company programs. 

## Discussion

We found a substantial increase in the use of best EBPs by the 17 companies that completed baseline and follow-up measurements in The CEOs Challenge in its first year. At baseline, implementation scores were lower for practices that promote healthy eating and cancer screening than for practices that promote physical activity or tobacco cessation. Healthy eating and cancer screening practices had the greatest improvement at 1-year follow-up. At both baseline and follow-up, all practice categories had room for improvement, even among these relatively large companies that were interested enough in health promotion to actively partner with the ACS. The participating companies were positive about the program and were poised for further participation.

We intended this evaluation as a feasibility-oriented pilot study rather than a trial of The CEOs Challenge. A limitation of the evaluation is its pre–post design and lack of a comparison group. A second limitation is that several participating companies were represented by local or regional offices rather than national headquarters offices, and local knowledge of the workplace-health–promotion practices of the national company may have been incomplete. Third, power for statistical testing may have been limited because of the small sample size. The distribution of change in overall company score from baseline to 12-month follow-up was somewhat skewed to the right, potentially violating the assumptions for the paired *t* test. However, the results from a signed rank test were confirmatory, and *t* test results were retained for greater statistical power. Additional *t* tests on the changes in the health-related best-practice categories were not conducted because of limitations of the sample; multiple tests could not be appropriately adjusted without losing so much power as to render the tests unreliable. Fourth, all practices were assessed by company managers and reflect their best knowledge, but the data may be incomplete.

The CEOs Challenge model also has several strengths. First, it addresses the 3 leading behavioral causes of death in the United States — tobacco use, physical inactivity, and unhealthy eating (11). So, success could have a substantial impact on both illness and death rates of employees of the participating companies. Second, the program targets large employers, which employ half of US workers (12). So, implementing The CEOs Challenge via the CEOs Against Cancer network, with its national breadth, could have high levels of reach among employees. Third, the program is largely oriented toward changing policy and environment. Policy and environmental changes are among the most efficient ways to improve health (13). Fourth, both at baseline and at follow-up, most of the participating employers had room for improvement on implementation of health-related best practices. These practices are evidence-based, but our baseline results show they are far from ubiquitous, even among these large employers. Fifth, ACS has a large, nationally distributed workforce engaged with 16 other CEOs Against Cancer chapters, each of which could implement The CEOs Challenge in its region.

The CEOs Challenge had little effect on the participating employers’ corporate philanthropy for ACS, at least as we measured it. Readers may question the appropriateness of including philanthropy as an explicit part of The CEOs Challenge. Public–private partnerships to improve the public’s health are most durable and effective when they address the needs of all partners (8). ACS, a nonprofit organization, raises funds through donations. The CEOs Challenge does present a potential conflict of interest for ACS because it asks employers to take sometimes-difficult policy and environmental approaches to improving employee health when it also asks them to assist with philanthropy. On the other hand, this group of employers was already engaged in corporate philanthropy and may have viewed The CEOs Challenge as ACS providing a service to their workplace and employees.

The CEOs Challenge is a promising approach to chronic disease prevention in the workplace. It brings together one of the nation’s largest health-promoting voluntary agencies with the nation’s largest employers to promote EBPs targeted at the most common causes of disease and death. The program appears to have increased the adoption of these practices and to have been well-accepted in one local chapter, and additional chapters could implement this approach or support a larger study of The CEOs Challenge with a comparison group. Both broader implementation and a larger study have the potential to significantly improve evidence-based health promotion opportunities for thousands of US employees and inform the science of organization-level adoption and implementation of EBPs.
